# A network pharmacology-based investigation on the bioactive ingredients and molecular mechanisms of *Gelsemium elegans Benth* against colorectal cancer

**DOI:** 10.1186/s12906-021-03273-7

**Published:** 2021-03-20

**Authors:** Wancai Que, Maohua Chen, Ling Yang, Bingqing Zhang, Zhichang Zhao, Maobai Liu, Yu Cheng, Hongqiang Qiu

**Affiliations:** 1 Department of Pharmacy, Fujian Medical University Union Hospital, 29 Xin Quan Rd, Gulou, Fuzhou, 350001 Fujian People’s Republic of China; 2 Department of Pharmacy, Fujian Cancer Hospital & Fujian Medical University Cancer Hospital, Fuzhou, China; 3 College of Pharmacy, Fujian Medical University, Fuzhou, 350004 People’s Republic of China

**Keywords:** *Gelsemium elegans Benth*, Colorectal cancer, Network pharmacology, Mechanism

## Abstract

**Background:**

Colorectal cancer (CRC) remains one of the leading causes of cancer-related death worldwide. *Gelsemium elegans Benth* (GEB) is a traditional Chinese medicine commonly used for treatment for gastrointestinal cancer, including CRC. However, the underlying active ingredients and mechanism remain unknown. This study aims to explore the active components and the functional mechanisms of GEB in treating CRC by network pharmacology-based approaches.

**Methods:**

Candidate compounds of GEB were collected from the Traditional Chinese Medicine@Taiwan, Traditional Chinese Medicines Integrated Database, Bioinformatics Analysis Tool for Molecular mechanism of Traditional Chinese Medicine, and published literature. Potentially active targets of compounds in GEB were retrieved from SwissTargetPrediction databases. Keywords “colorectal cancer”, “rectal cancer” and “colon cancer” were used as keywords to search for related targets of CRC from the GeneCards database, then the overlapped targets of compounds and CRC were further intersected with CRC related genes from the TCGA database. The Cytoscape was applied to construct a graph of visualized compound-target and pathway networks. Protein-protein interaction networks were constructed by using STRING database. The DAVID tool was applied to carry out Gene Ontology and Kyoto Encyclopedia of Genes and Genome pathway enrichment analysis of final targets. Molecular docking was employed to validate the interaction between compounds and targets. AutoDockTools was used to construct docking grid box for each target. Docking and molecular dynamics simulation were performed by Autodock Vina and Gromacs software, respectively.

**Results:**

Fifty-three bioactive compounds were successfully identified, corresponding to 136 targets that were screened out for the treatment of CRC. Functional enrichment analysis suggested that GEB exerted its pharmacological effects against CRC via modulating multiple pathways, such as pathways in cancer, cell cycle, and colorectal cancer. Molecular docking analysis showed that the representative compounds had good affinity with the key targets. Molecular dynamics simulation indicated that the best hit molecules formed a stable protein-ligand complex.

**Conclusion:**

This network pharmacology study revealed the multiple ingredients, targets, and pathways synergistically involved in the anti-CRC effect of GEB, which will enhance our understanding of the potential molecular mechanism of GEB in treatment for CRC and lay a foundation for further experimental research.

**Supplementary Information:**

The online version contains supplementary material available at 10.1186/s12906-021-03273-7.

## Background

Colorectal cancer (CRC) continues to be one of the leading causes of mortality and morbidity worldwide despite the availability of reliable screening tools and effective therapies. It is the second most common cause of cancer death in the United States when men and women are combined [1]. According to the American Cancer Society’s and GLOBOCAN estimates, it will be 147,950 and 1,931,590 new cases of CRC in the United States and the world for 2020, respectively [2, 3]. The incidence and mortality of CRC are rapidly increasing particularly in developing countries, and it is estimated that the global burden of CRC increases by 60% over 2.2 million new cases and 1.1 million cancer death by 2030 [4]. Effective treatments used for CRC may include some combination of surgery, radiation therapy, chemotherapy, immunotherapy and targeted therapy [5–7]. However, the mortality is still relatively high because of delayed diagnosis, metastasis, and frequent recurrence. The 5-year survival rate is less than14% and unfortunately, more than 50% of CRC patients were diagnosed at an advanced stage [8]. Furthermore, using the most prevalent chemotherapy regimens has shown the limitations, a series of side effects commonly accompany patients, such as gastrointestinal reaction, bone marrow suppression, neurotoxicity, and abnormal liver or kidney function. It is of great significance to search for more effective alternative agents with low toxicity for patients.

Medicinal herbs are an important, yet often overlooked, a source for novel antineoplastic drugs. Indeed, many chemotherapeutics derived from plants, such as paclitaxel, vinblastine, and vincristine, have proven effective against different tumors. Sometimes as a complementary therapy, medicinal plants are widely used to treat several types of cancers, including CRC, with relatively fewer and milder side effects [9, 10]. As an important source of alternative and complementary medicines, traditional Chinese medicine (TCM) has been widely reported to treat cancer [11–13]. In recent years, more and more herbs originating from TCM have attracted considerable attention as anti-CRC agents because of their therapeutic value and low toxicity [9, 14].

**Fig. 1:**
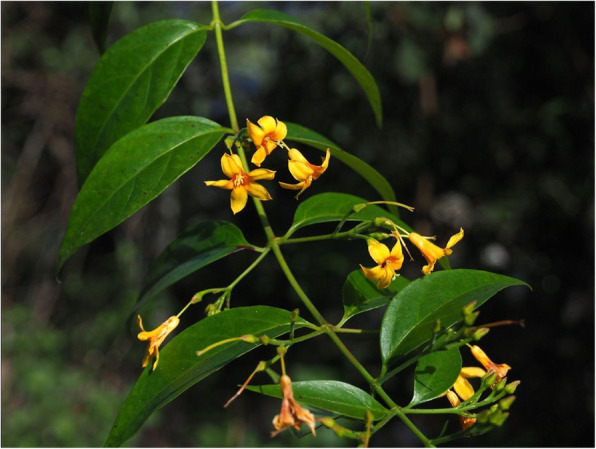
Flowers and leaves from *Gelsemium elegans Benth*

*Gelsemium*, as a genus of the *Gelsemiaceae* family, consists of three well-known species: *Gelsemium elegans Benth*. (GEB) (Fig. 1), native to Southeast Asia and China, and *Gelsemium rankinii Small* and *Gelsemium sempervirens Ait*, native to North America [15]. Although GEB is a toxic plant, it has long been used in Chinese folk medicine to treat many diseases, including pain, inflammation, and cancer [16, 17]. Alkaloids, isolated and purified from GEB, constitute the main active molecules of GEB and were profoundly studied for their biological activities in several pharmaceutical areas, including anti-inflammatory, antirheumatic, analgesic, immunomodulatory, and anti-tumor activities [16]. GEB has shown potential as a promising anti-tumor agent in clinical practice. Patients with severe primary liver cancer survived after treated with powder of GEB (150 mg,Bid), resuting in tumor shrinkage and pain relief [18]. Notably, GEB has long been used as Chinese folk medicine in the treatment of CRC in Southern China [19]. In vitro study, cytotoxic effects on the tumor cells of digestive system was observed in alkaloidal compounds from GEB [20]. Alkaloids of GEB could inhibit the proliferation and induced the apoptosis of the human colonic carcinoma cells [21, 22]. However, the potential active compounds and underlying molecular mechanisms of the anti-CRC effect of GEB remain unknown.

With the current merging of bioinformatics, network pharmacology-based analysis has become a robust method to systematically reveal the biological mechanisms of complex diseases and drug effects at the molecular level [23]. It integrates information science, systematic medicine, and is evolving as a promising strategy for the next-generation mode of drug discovery and development for traditional medicine. Compared with traditionally experimental pharmacology research, network pharmacology focuses on analyzing multiple target regulation of multiple chemical components, so it is particularly suitable for the interpretation of the mechanism of TCM [24]. Hence, the present study aimed to reveal the potentially active ingredients against CRC and predict the underlying action mechanism of GEB by employing a network pharmacological method.

## Methods

### Potentially active compounds in GEB

The information about compounds of GEB was obtained from the published literature [16, 25] and the following online databases: (1) Traditional Chinese Medicines Integrated Database (TCMID) (http://119.3.41.228:8000/tcmid/), which is a comprehensive database to provide information and bridge the gap between TCM and modern life sciences [26]. (2) Bioinformatics Analysis Tool for Molecular mechanism of Traditional Chinese Medicine (BATMAN-TCM) (http://bionet.ncpsb.org/batman-tcm/), which is the first online bioinformatics analysis tool specially designed for the research of molecular mechanism of TCM [27]. (3) Traditional Chinese Medicine Database@ Taiwan (http://tcm.cmu.edu.tw/zh-tw/), the world’s largest TCM database for drug screening in silico [28]. Then all compounds of the herbal medicine were determined by removing the duplicate compounds.

The candidate active compounds were further filtered by meeting at least two of five features of drug-likeness (Lipinski, Ghose, Veber, Egan, and Muegge) and combining bioavailability score ≥ 30% as suggested by the http://www.swissadme.chwebsite, which allows to compute physicochemical descriptors as well as to predict ADME parameters, pharmacokinetic properties, druglike nature and medicinal chemistry friendliness of one or multiple small molecules to support drug discovery [29].

### Targets prediction of compounds in GEB

As a popular online server, SwissTargetPrediction (http://www.swisstargetprediction.ch) provides information on chemical substances, biological activities, and allows to estimate the most probable macromolecular targets of a small molecule [30]. 3D molecular structure files of each ingredient that could be retrieved from PubChem (https://pubchem.ncbi.nlm.nih.gov/) were imported into SwissTargetPrediction for identification of potential drug target in humans. The targets of ingredients acquired from SwissTargetPrediction with probability ≥0.1 were chosen as potential targets in this study after removing the repeated targets. Those compounds without target information were excluded.

### Targets of CRC

Information on CRC-associated target genes was collected from the following resources. The different genes involved in CRC were gathered from GeneCards (https://www.genecards.org/), which is a searchable, integrative database that provides comprehensive, user-friendly information on all annotated and predicted genes involved in human diseases [31]. Keywords “colorectal cancer”, “rectal cancer”, and “colon cancer”, were used to search through the database, which identified 33,505 genes with a disease relevance score ≥ 10. Then, the putative target genes of GEB were mapped to the CRC-associated target genes. The candidate anti-CRC targets of GEB were visualized by overlapping the above targets with a Venn diagram.

### Validation of candidate targets in the cancer genome atlas (TCGA) database

CRC related genes from The Cancer Genome Atlas (TCGA) database of ONCOMINE (https://www.oncomine.org/) were used to validate candidate targets of GEB against CRC. Firstly, the top 10% over-expression and under-expression mRNA genes form CRC samples (Cecum Adenocarcinoma vs. Normal, Colon Adenocarcinoma vs. Normal, Colon Mucinous Adenocarcinoma vs. Normal, Rectal Adenocarcinoma vs. Normal, Rectal Mucinous Adenocarcinoma vs. Normal, and Rectosigmoid Adenocarcinoma vs. Normal) were downloaded from the TCGA database of ONCOMINE. Subsequently, a Venn diagram was used to intersect the candidate targets of GEB against CRC genes obtained from the TCGA database.

### Protein-protein interaction (PPI) data

Core regulatory genes can be identified by exploring the protein-protein interaction (PPI). PPI information can be obtained from the STRING database (https://string-db.org/), which covers abundant information regarding known and predicted protein-protein interactions of different species [32]. In this study, high confidence score > 0.7 were reserved and the species was only limited to “*Homo sapiens*”, then the validated targets were submitted to STRING. Finally, PPI data were extracted. The top 20 proteins with a higher level of degrees were considered as the center targets for GEB in the treatment for CRC.

### Cluster analysis

There are some closely connected regions of molecular complexes in large PPI networks, which are named topology modules or clusters. Cluster analysis is a classification method that involves interconnected regions showing the inherent laws in the network. In this study, significant cluster modules from the constructed PPI network were selected using the Molecular Complex Detection (MCODE), a plug-in of Cytoscape, which was used to detect densely connected regions and cluster analysis in the PPI network [33]. The criteria settings were set as follows: node score cutoff = 0.2; K-core = 2; and degree of cutoff = 2 [34].

### GO and KEGG pathway enrichment analysis

To explore the gene functions, the Database for Annotation, Visualization and Integrated Discovery (DAVID, https://david.ncifcrf.gov/.ver.6.8) which provides a systematic and comprehensive set of functional annotation tools for investigators to understand the biological meanings behind a large list of genes [35], was applied to perform Gene Ontology (GO) and Kyoto encyclopedia of genes and genome (KEGG) pathway enrichment of proteins in the PPI network analyses.The species was only limited to “*Homo sapiens*”. Those GO and KEGG pathway terms with only False Discovery Rate (FDR) < 0.01 were considered to be significantly enriched. As for enrichment analysis, the results of enriched GO terms of biological process (BP), cellular component (CC), and molecular function (MF) were visualized by the R software package (3.5.2), as well as the bubble chart of KEGG pathway enrichment.

### Network construction

Four networks were constructed as follows: (1) Compounds-compound targets network of GEB was constructed by connecting chemical compounds with corresponding targets; (2) Potential compounds-targets network of GEB against CRC; (3) Potential compounds-targets-pathways network of GEB against CRC; (4) PPI network of the potential targets of GEB against CRC. The PPI network was completed directly on STRING. The other 3 networks were constructed using the network visualization software Cytoscape (http://cytoscape.org/.ver.3.7.2), which is an open-source software platform suitable for visualizing intermolecular interactions networks and biological pathways [36]. Furthermore, Cytoscape can be used to integrate and analyze these networks with annotations, gene expression profiles, and other complicated data. Three parameters can be calculated to evaluate the topological coefficients of each node. “Degree” represents the number of edges connected to a node; “Betweenness” is defined as the number of times a node act as a bridge along the shortest paths between pairs of other nodes; “Closeness” is the inverse of the sum of the shortest paths from a node to other nodes in the network.

### Active compounds-targets docking

Ten compounds were selected from the core compounds of GEB and docked with six proteins selected from the center targets to verify the accuracy of the main compounds and their corresponding predicted targets. The candidate compound and the crystal structure of the target protein were downloaded from the PubChem database and RCSB protein data (http://www.rcsb.org), respectively. The latter preferably selects a model with ligand binding smaller than 3 Å, and then dehydration, hydrogenation, and separation of ligands were carried out by importing the crystal structure into the Pymol 2.4.1 Software (https://pymol.org/2/); then AutoDockTools 1.5.6 was used to construct the docking grid box of crystal structure for each target [37]. Docking was done by Autodock Vina 1.1.2 software, and the molecules with the lowest binding energy in the docking conformation were selected to observe the binding effect by comparing with the original ligands and intermolecular interactions (such as hydrophobicity, cation-π, anion-π, π-π stacking, hydrogen bonding, etc.). The proteins with the original ligands were specified docking at a domain of the protein, and amino acid residues in the domain were targeted for evaluating the interaction. The number of grid points in the three dimensions (NPTS) used in this study were 40 40 40 0.375. Since RCSB did not find the effective crystal structure of the CCND1 binding ligand, direct docking was performed with grid center 24.683 13.205 61.426.

### Molecular dynamics simulation

In order to analyze the binding affinities of the best hit molecules (gelsesyringalidine and CDK2) after docking, a 10 ns atomistic molecular dynamics (MD) simulation of selected protein-ligand complex was conducted. In the present study the NVIDIA RTX 1060 GPU accelerated GROMACS 2021 software, running over Linux ubuntu 20.04 operating system supported by AMD R5 3600 processor was used. The Charmm36 force field was used to generate protein topology. The ligand topology and parameters required for MD simulation were generated by using CGenFF server. The TIP3P water model was used for solvating each systems followed by neutralization with appropriate numbers of Na^+^ and Cl^−^. Then energy of each system was minimized by using the steepest descent minimization algorithm with maximum 50,000 steps and < 10.0 kJ/mol force. Position restrains have been applied to receptor and ligand of the each systems for 100 ps throughout heating (300 K) utilizing NVT (No. of atoms, Volume, Temperature) ensemble with leap-frog integrator, a time step of 2 fs and LINCS holonomic constraints.NPT (No. of atoms, Pressure, Temperature) ensemble has been applied at temperature (300 K) for 100 ps using a time step of 2 fs for NPT equilibration phase. After the energy minimization and equilibration of all systems, MD production run has been executed without any restrain for 10 ns with a time step of 2 fs, and after every 10 ps coordinates of the structure have been saved. After the completion of 10 ns MD simulation, the trajectories have been used for various dynamics analysis such as root mean square deviation (RMSD) and root mean square fluctuation (RMSF). These were compared with the primitive ligand complex.

### Statistics

Benjamini–Hochberg correction was performed for multiple testing, and adjusted value <0.05 was set as the threshold. False Discovery Rate (FDR) < 0.01 was deemed as significant enriched in GO and KEGG analysis.

## Results

### Potentially active compounds and targets in GEB

**Fig. 2:**
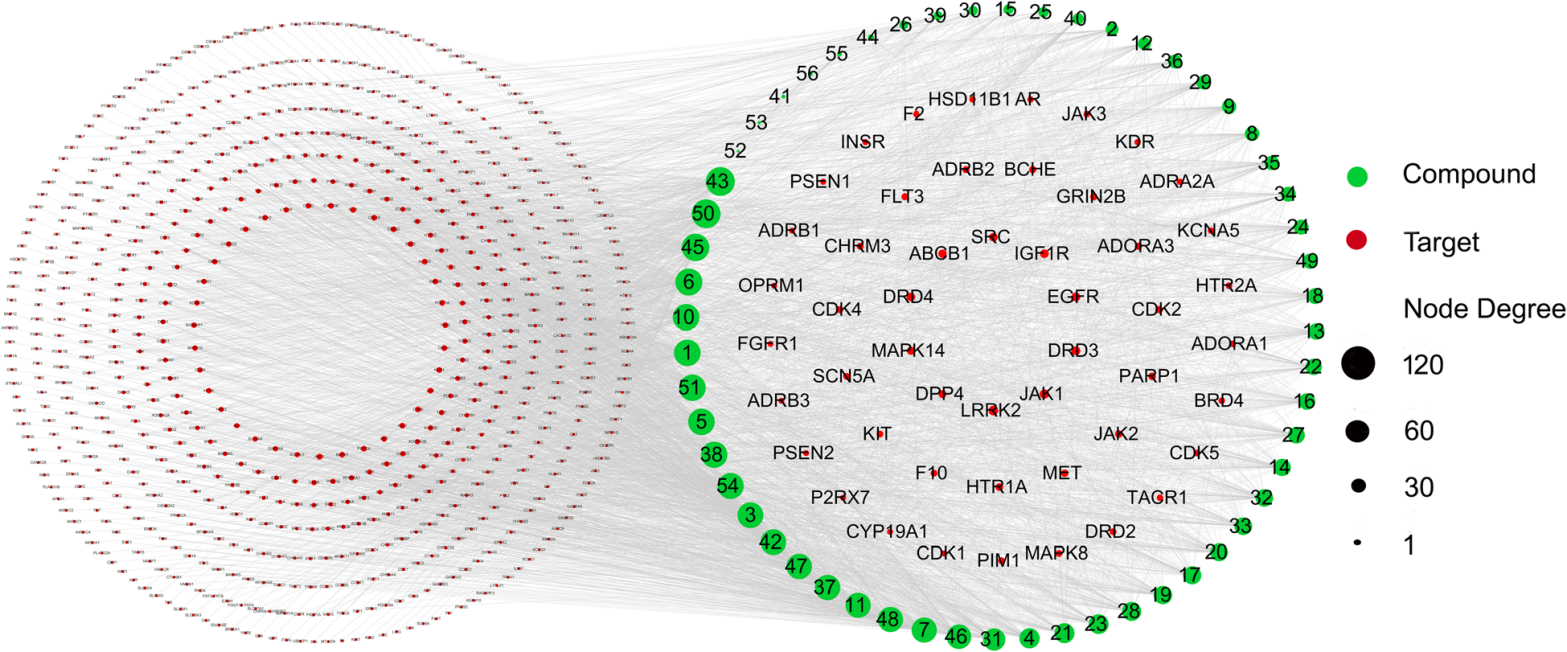
Compounds-targets network of GEB

Using the BATMAN-TCM and TCM@Taiwan databases, we collected a total of 97 compounds in GEB. Eventually, based on the filtering rules, (OB ≥30% and the features of drug-likeness), 56 potentially active compounds were identified from a total compound in GEB. Details of the 56 potentially bioactive compounds are provided in Table 1. By using SwissTargetPrediction for target prediction, 729 potential targets were found for GEB (Table S1). The compounds-compound targets network as shown in Fig. 2, included 785 nodes: 56 active compound nodes and 729 target nodes, and 3747 edges. The top 5 compound nodes with the greatest number of edges included Humantenine, n-desmethoxtrankinidine, 19-(Z)-Akuammidine, 19 - Z- akuammidine, and N- Desmethoxyhumantenine. Three topological features of these compounds exhibited mean values of degree, node betweenness, and closeness were 111.2, 0.04769 and 0.3723, respectively. The top 5 target nodes with highest degree were LRRK2, JAK1, DRD3, EGFR, and IGF1R. nidine, 19-(Z)-Akuammidine, 19 - Z- akuammidine, and N- Desmethoxyhumantenine. Three topological features of these targets exhibited mean values of degree, node betweenness, and closeness were 29, 0.01554 and 0.4025, respectively.

**Table 1:** Information of the active compounds in GEB for network analysis

NO	Name	Compound CID	MW	MF	Source
1	N- Desmethoxyrankinidine	5,316,594	310.4	C_19_H_22_N_2_O_2_	TCMID, References [16, 24]
2	11- Methoxygelsemamide	5,319,437	355.4	C_21_H_25_NO_4_	TCM-Taiwan, References [16, 24]
3	Gelsevirine	14,217,344	352.4	C_21_H_24_N_2_O_3_	TCMID, BATMAN-TCM, TCM-Taiwan, References [16, 24]
4	Gelsenicine	21,123,652	326.4	C_19_H_22_N_2_O_3_	References [16, 24]
5	Gelsedine	21,589,070	328.4	C_19_H_24_N_2_O_3_	TCMID, BATMAN-TCM, TCM-Taiwan, References [16, 24]
6	19 - Z- akuammidine	44,583,830	352.4	C_21_H_24_N_2_O_3_	References [16, 24]
7	Dihydrokoumine	5,316,727	308.4	C_20_H_24_N_2_O	BATMAN-TCM, References [16, 24]
8	19- (R)- Hydroxydihydrokoumine	50,278,496	324.4	C_20_H_24_N_2_O_2_	TCMID, BATMAN-TCM, TCM-Taiwan, References [16, 24]
9	19- (S)- Hydroxydihydrokoumine	5,318,193	324.4	C_20_H_24_N_2_O_2_	References [16, 24]
10	N- Desmethoxyhumantenine	5,316,593	324.4	C_20_H_24_N_2_O_2_	TCMID, BATMAN-TCM, Reference [24]
11	15- Hydroxyhumantenine	101,606,434	370.4	C_21_H_26_N_2_O_4_	TCMID, References [16, 24]
12	Humantenmine	158,212	326.4	C_19_H_22_N_2_O_3_	TCMID, BATMAN-TCM, TCM-Taiwan, Reference [24]
13	11-Hydroxyrankinidine	5,318,332	356.4	C_20_H_24_N_2_O_4_	TCMID, References [16, 24]
14	11-Hydroxyhumantenine	5,318,224	370.4	C_21_H_26_N_2_O_4_	TCMID, References [16, 24]
15	11-Methoxyhumantenine	44,583,832	384.5	C_22_H_28_N_2_O_4_	TCMID, BATMAN-TCM, References [16, 24]
16	19- (S)- Hydroxydihydrogelsevirine	5,318,192	370.4	C_21_H_26_N_2_O_4_	References [16, 24]
17	Gelseoxazolidinine	102,297,300	428.5	C_23_H_28_N_2_O_6_	References [16, 24]
18	Gelselegine	10,948,335	358.4	C_20_H_26_N_2_O_4_	TCMID, References [16, 24]
19	19α-Hydroxygelsamydine	102,003,053	524.6	C_29_H_36_N_2_O_7_	References [16, 24]
20	Gelsamydine	5,317,540	508.6	C_29_H_36_N_2_O_6_	TCMID, References [16, 24]
21	Gelegamine E	101,467,881	370.4	C_20_H_22_N_2_O_5_	References [16, 24]
22	Gelegamine A	101,467,877	384.4	C_21_H_24_N_2_O_5_	References [16, 24]
23	Gelegamine B	101,467,878	384.4	C_21_H_24_N_2_O_5_	References [16, 24]
24	Gelsenine	NA	358.19	C_20_H_26_N_2_O_4_	References [16, 24]
25	21- Oxokoumine	NA	320.1	C_20_H_20_N_2_O_2_	References [16, 24]
26	Furanokoumine	NA	322.1	C_20_H_22_N_2_O_2_	References [16, 24]
27	Koumidine	44,584,550	294.4	C_19_H_22_N_2_O	TCMID, BATMAN-TCM, References [16, 24]
28	Gelebolines A	NA	320.15	C_20_H_20_N_2_O_2_	References [16, 24]
29	19- Z- taberpsychine	5,321,582	310.4	C_20_H_26_N_2_O	References [16, 24]
30	Nb-Methylgelsedilam	NA	328.14	C_18_H_20_N_2_O_4_	References [16, 24]
31	Gelsesyringalidine	136,704,418	490.5	C_28_H_30_N_2_O_6_	References [16, 24]
32	14- Dehydroxygelsefuranidine	102,417,029	404.5	C_24_H_24_N_2_O_4_	References [16, 24]
33	Humantenoxenine	NA	368.17	C_21_H_24_N_2_O_4_	References [16, 24]
34	Kounaminal	102,260,292	363.5	C_22_H_25_N_3_O_2_	References [16, 24]
35	Dehydrokoumidine	119,077,162	292.4	C_19_H_20_N_2_O	References [16, 24]
36	Koumine	91,895,267	306.4	C_20_H_22_N_2_O	TCMID, BATMAN-TCM, TCM-Taiwan, References [16, 24]
37	Gelsemine	279,057	322.4	C_20_H_22_N_2_O_2_	TCMID, BATMAN-TCM, Reference [16]
38	Elegansamine	5,317,023	508.6	C_29_H_36_N_2_O_6_	TCMID, BATMAN-TCM
39	Ranol	10,939,065	438.6	C_26_H_46_O_5_	TCMID, BATMAN-TCM, TCM-Taiwan
40	12α-Hudroxyrotenone	161,176	410.4	C_23_H_22_O_7_	TCMID, TCM-Taiwan
41	5-methoxy-7-(4-hydroxy-33methoxy phenyl)-1-phenyl-3-heptanone	26,088,037	342.4	C_21_H_26_O_4_	TCMID, TCM-Taiwan
42	15-Hydroxyhumamtenine	101,606,434	370.4	C_21_H_26_N_2_O_4_	TCMID
43	Humantenine	44,593,672	354.4	C_21_H_26_N_2_O3	TCMID, BATMAN-TCM, TCM-Taiwan
44	Akuammidine N-Oxide	11,268,654	368.4	C_21_H_24_N_2_O_4_	TCMID, BATMAN-TCM, TCM-Taiwan
45	19-(Z)-Akuammidine	44,583,830	352.4	C_21_H_24_N_2_O_3_	TCMID, TCM-Taiwan
46	Tabersonine	20,485	336.4	C_21_H_24_N_2_O_2_	TCMID, BATMAN-TCM, TCM-Taiwan
47	Gelsemicine	5,462,428	358.4	C_20_H_26_N_2_O_4_	TCMID, BATMAN-TCM, TCM-Taiwan
48	19-(Z)-Taberpsychine	5,321,582	310.4	C_20_H_26_N_2_O	TCMID, BATMAN-TCM
49	19-(S)-Hydroxydihydrokoumine	5,318,193	324.4	C_20_H_24_N_2_O_2_	TCMID, TCM-Taiwan
50	n-desmethoxtrankinidine	5,316,593	324.4	C_20_H_24_N_2_O_2_	TCMID
51	Oxoglaucine	97,662	351.4	C_20_H_17_NO_5_	TCMID, BATMAN-TCM, TCM-Taiwan
52	Sempervirine (II)	168,919	272.3	C_19_H_16_N_2_	TCMID, TCM-Taiwan
53	6-methoxygeniposidic acid	6,325,623	404.4	C_17_H_24_O_11_	TCMID
54	hydroxygenkwanin	5,318,214	300.3	C_16_H_12_O_6_	TCMID
55	dihydromelilotoside	5,316,728	328.3	C_15_H_20_O_8_	TCMID
56	7-hydroxydihydromatatabiether	5,318,195	170.4	C_10_H_18_O_2_	TCMID

NA: Not applicable

Red circle nodes stand for the target genes. Green nodes stand for compounds in GEB.

The size of the node represents the number of the degrees.

### Targets of compounds in GEB against CRC

**Fig. 3:**
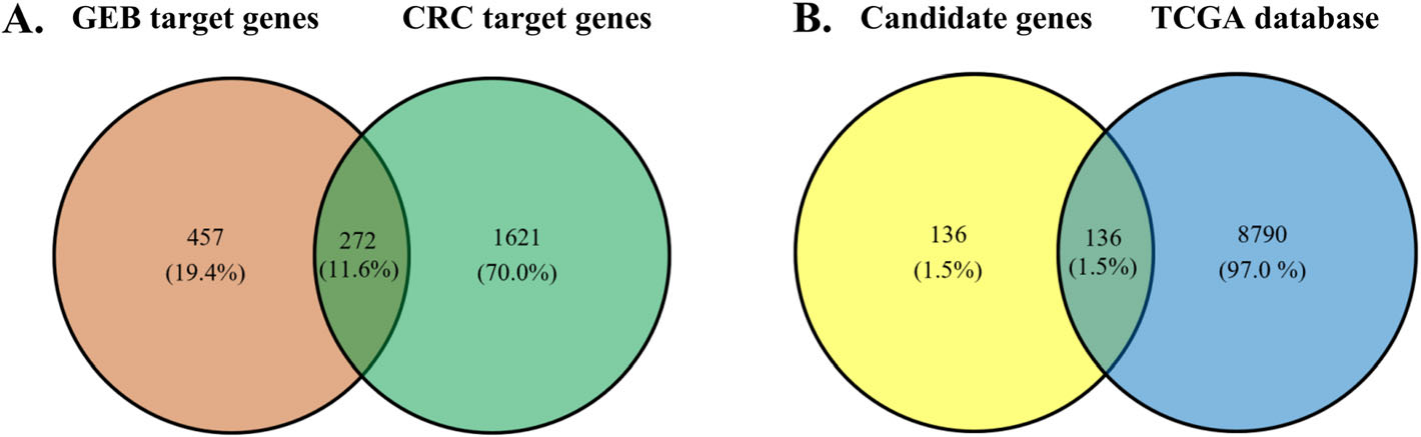
GEB target genes prediction for CRC treatment

CRC-related target genes were retrieved from GeneCards, 33,505 potential target genes related to CRC were obtained. A total of 1893 potential target genes were included as candidate genes with a disease relevance score ≥ 10. The details about the selected 1893 candidate target genes are described in Table S2. Then, the predictive target genes of GEB were overlapped with the candidate target genes. By intersecting the 729 target genes of GEB with the 1893 candidate target genes related to CRC, we obtained 272 intersections of target genes excluding any duplicate targets (Table S3, Fig. 3A). These intersections were considered potential candidate targets of GEB against CRC.

The overlapping number of GEB target genes and CRC (A), and the number of overlapping GEB candidate genes against CRC and CRC genes from The Cancer Genome Atlas (TCGA) database (B).

### Candidate therapeutic targets validation in the TCGA database

**Fig. 4:**
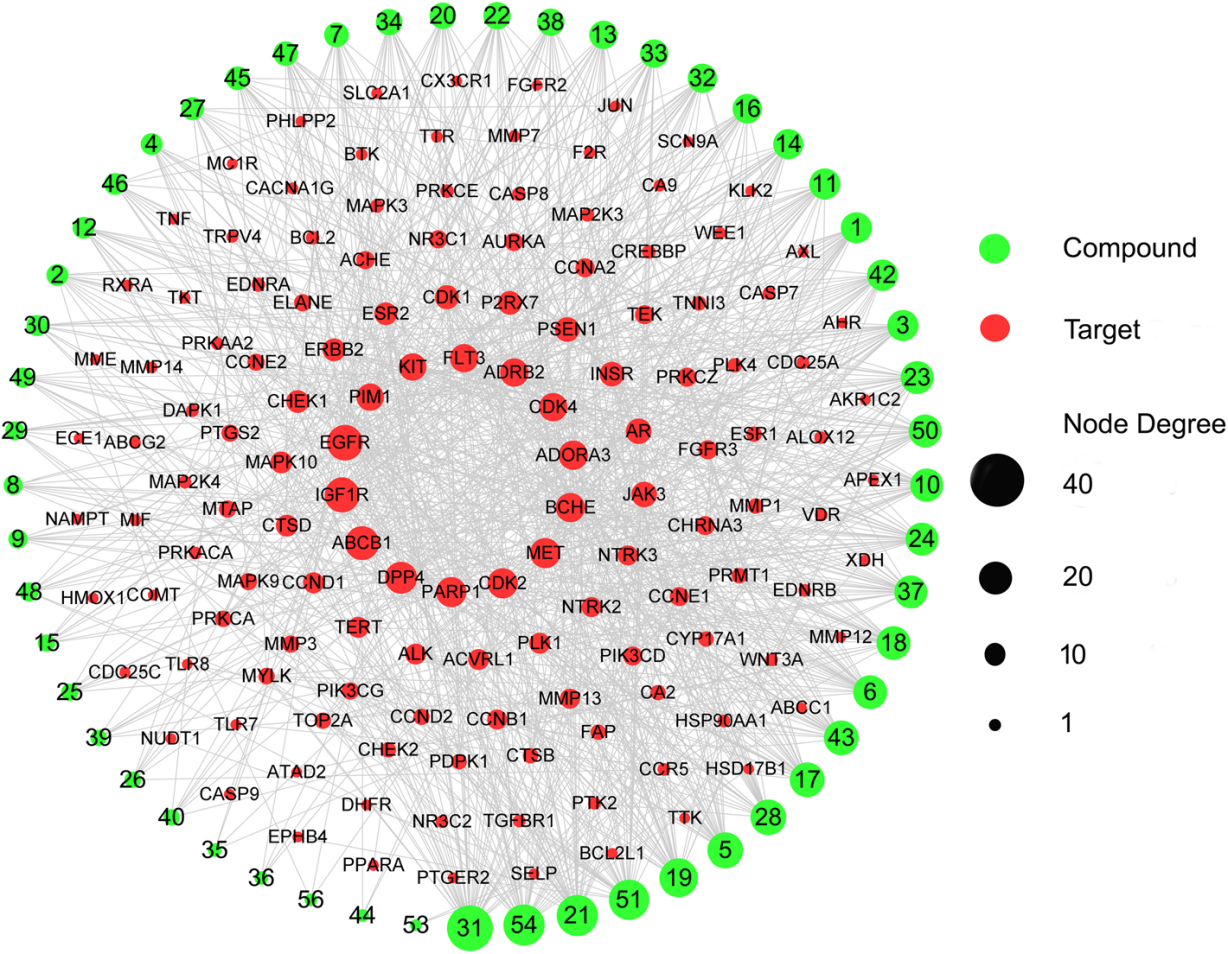
Compounds-potential targets network of GEB in treatment for CRC

A total of 8926 CRC related genes were identified in 215 tumor samples and 22 adjacent non-tumor samples from the TCGA database. These genes were significantly over-expressed and under-expressed genes (Table S4). Comparison of the 272 potential GEB candidate target genes against CRC with the 8926 CRC related genes from TCGA revealed 136 common genes (Fig. 3B). These 136 common genes were considered key potential anti-CRC target genes of GEB (Table S5). As shown in Fig. 4, the compounds and targets related to CRC network analysis indicated that the top 5 compound nodes with the greatest number of edges included gelsesyringalidine, hydroxygenkwanin, Gelegamine E, oxoglaucine, and 19α-hydroxygelsamydine. Three topological features of these compounds exhibited mean values of degree, node betweenness, and closeness were 34.2, 0.08659 and 0.4206, respectively. The top 5 gene nodes with the greatest number of edges included EGFR, IGF1R, ABCB1, DPP4, and PARP1. Three topological features of these compounds exhibited mean values of degree, node betweenness, and closeness were 25.4, 0.03637 and 0.4359, respectively.

Red circle nodes stand for the potential target genes related to CRC. Green nodes stand for potential active compounds in GEB for treating CRC. The size of the node represents the number of the degrees.

### PPI network of targets for GEB against CRC

**Fig. 5:**
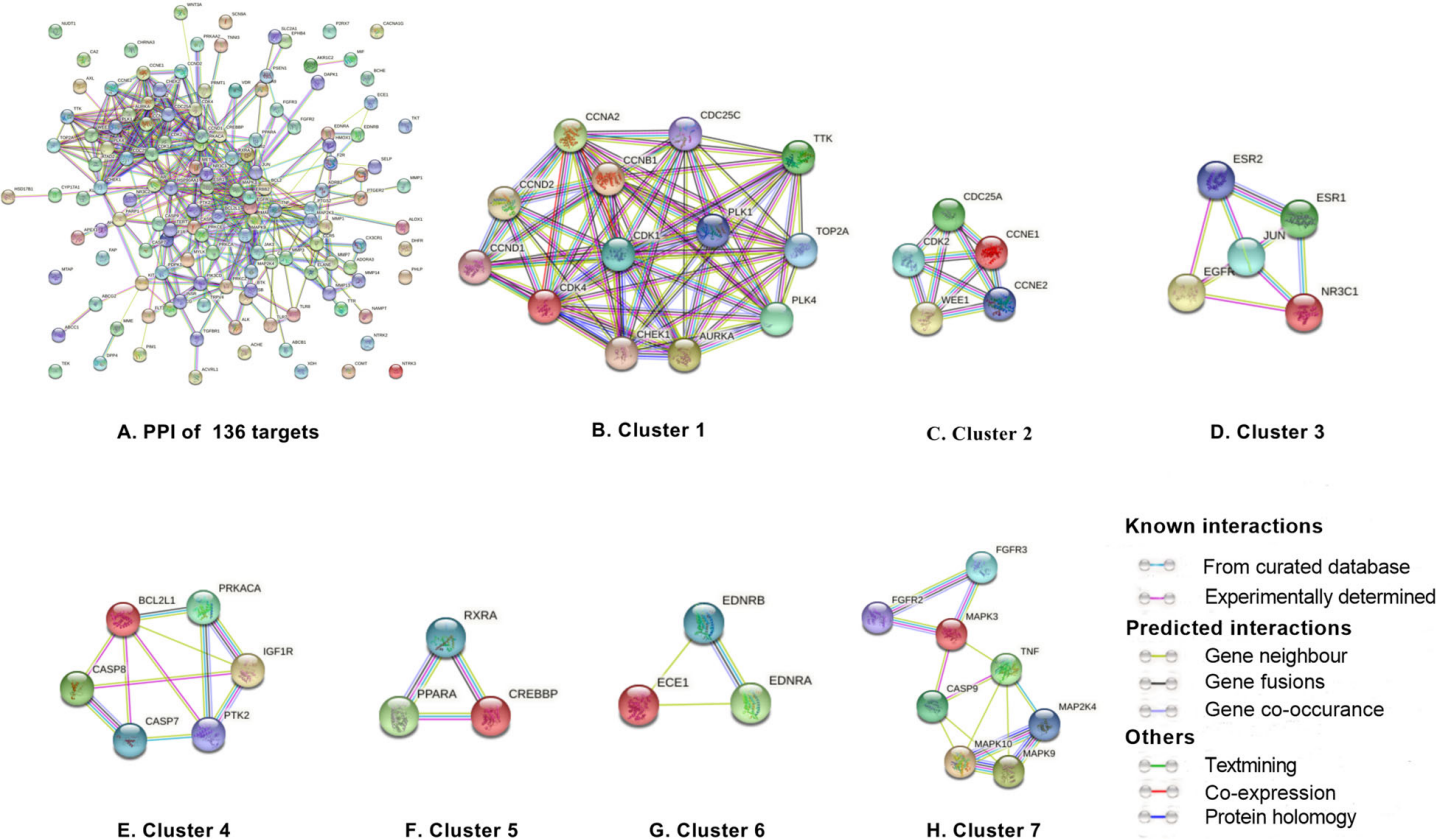
Protein-protein interaction network for GEB in treatment for CRC

To further identify the core regulatory targets of GEB against CRC, the STRING tool was employed to establish PPI network of the 136 targets. The PPI network of the potential targets of GEB against CRC was shown in Fig. 5A. With the confidence score > 0.7 was selected, the network of PPI was composed of 118 nodes and 458 edges. The top 20 proteins with higher levels of connectivity were selected as the center targets for GEB against CRC. The center targets, which may play a key role in GEB against CRC, namely, MAPK3, HSP90AA1, JUN, EGFR, CDK1, TNF, CCND1, ESR1, PRKACA, CCNA2, CDC25C, CDK2, CCNB1, AR, CREBBP, AURKA, CDC25A, CHEK1, BCL2L1, and PIK3CD. Three topological features of these top 20 targets exhibited mean values of degree, node betweenness, and closeness were 20.75, 0.05843 and 0.4442, respectively.

Protein-protein interaction of 136 potential targets (A) and 7 clusters (B, C, D, E, F, G, and H). Edges represent protein-protein associations, including known interaction (azure represents curated database evidence, purple represents experimentally determined evidence), predicted interactions (green represents gene neighborhood, red represents gene fusions, and blue represents gene co-occurrence), and others (light green represents text mining, black represents co-expression, and light blue represents protein homology).

### Cluster analysis

MCODE network analysis revealed 7 clusters (Fig. 5B, C, D, E, F, G, and H). The highest scoring cluster, cluster 1, contained 13 nodes and 58 edges, including CCND2, TOP2A, CDK4, TTK, PLK1, CCNA2, CCND1, CCNB1, PLK4, CDK1, CDC25C, CHEK1, and AURKA. Cluster 2 contained 5 nodes (CDK2, CDC25A, CCNE1, WEE1, and CCNE2) and 10 edges. Cluster 3 contained 5 nodes (JUN, ESR1, EGFR, NR3C1, and ESR2) and 8 edges. The detail of other clusters was shown in Table 2.

**Table 2:** Clusters of the protein-protein interaction (PPI) network

Cluster	Score	Nodes	Edges	Gene IDs
1	9.667	13	58	CCND2, TOP2A, CDK4, TTK, PLK1, CCNA2, CCND1, CCNB1, PLK4, CDK1, CDC25C, CHEK1, AURKA
2	5	5	10	CDK2, CDC25A, CCNE1, WEE1, CCNE2
3	4	5	8	JUN, ESR1, EGFR, NR3C1, ESR2
4	3.2	6	8	PTK2, IGF1R, CASP8, PRKACA, CASP7, BCL2L1
5	3	3	3	PPARA, CREBBP, RXRA
6	3	3	3	EDNRA, ECE1, EDNRB
7	2.857	8	10	MAPK3, MAP 2 K4, CASP9, FGFR3, FGFR2, MAPK9, TNF, MAPK10

### GO and KEGG pathway enrichment analysis

**Fig. 6:**
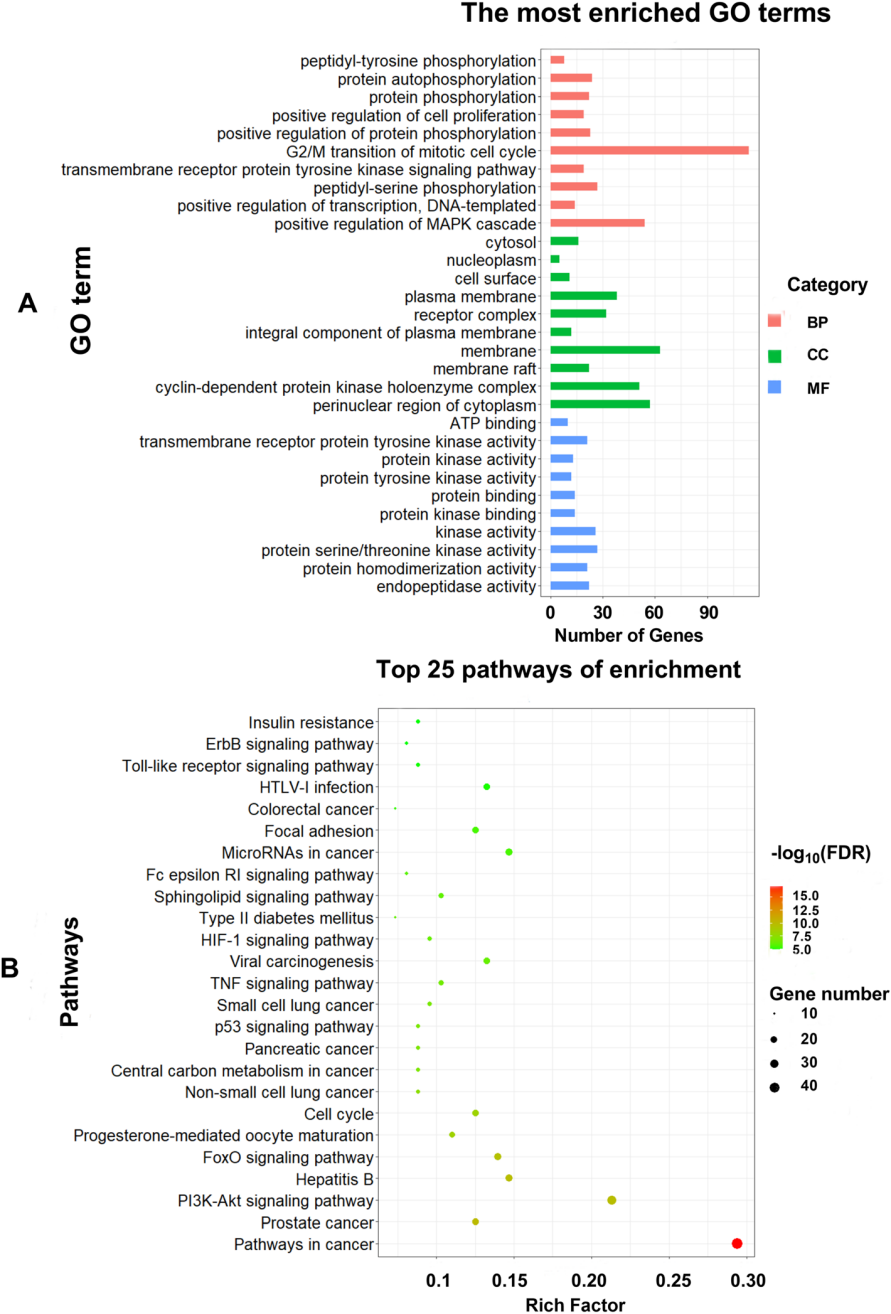
GO and KEGG pathway enrichment analysis of GEB in treatment for CRC

To explore the multiple functions of 136 potential anti-CRC targets of GEB, GO analysis and KEGG pathway enrichment of the candidate targets were performed. In GO analysis, the 136 potential target genes were significantly enriched in 70 biological process (BP), 22 cell components (CC), and 24 molecular functions (MF) (FDR < 0.01, Supplementary Table S6). The top 3 BP terms were peptidyl-tyrosine phosphorylation (GO: 0018108), protein autophosphorylation (GO: 0046777), and protein phosphorylation (GO: 0006468). The top 3 CC terms included cytosol (GO: 0005829), nucleoplasm (GO:0005654), and cell surface (GO:0009986). The top 3 MF terms were mainly enriched in ATP binding (GO:0005524), transmembrane receptor protein tyrosine kinase activity (GO:0004714), and protein kinase activity (GO: 0004672). KEGG pathway analysis identified 70 pathways that potentially participate in the anti-CRC of GEB. The top 3 pathways were pathways in cancer (hsa05200), prostate cancer (hsa05215), and PI3K-Akt signaling pathway (hsa04151). The pathway of colorectal cancer (hsa05210) was also remarkably enriched (FDR < 0.01, Supplementary Table S7). Top 10 GO functional categories in BP, CC, and MF, and 25 remarkable pathways were selected and were presented in Fig. 6.

The GO enrichment analysis of GEB anti-CRC genes (A). The ontology covered 3 domains: biological process, cellular component, and molecular function. The KEGG enrichment analysis of GEB against CRC related genes (B). The abscissa represents the rich factors, the proportion of genes enriched in the according to the pathway, the ordinate represents the pathway, and the color of the circle represents the corrected FDR-value. KEGG- Kyoto Encyclopedia of Genes and Genomes; GO- Gene Ontology.

### Compound-target-pathway network construction

**Fig. 7:**
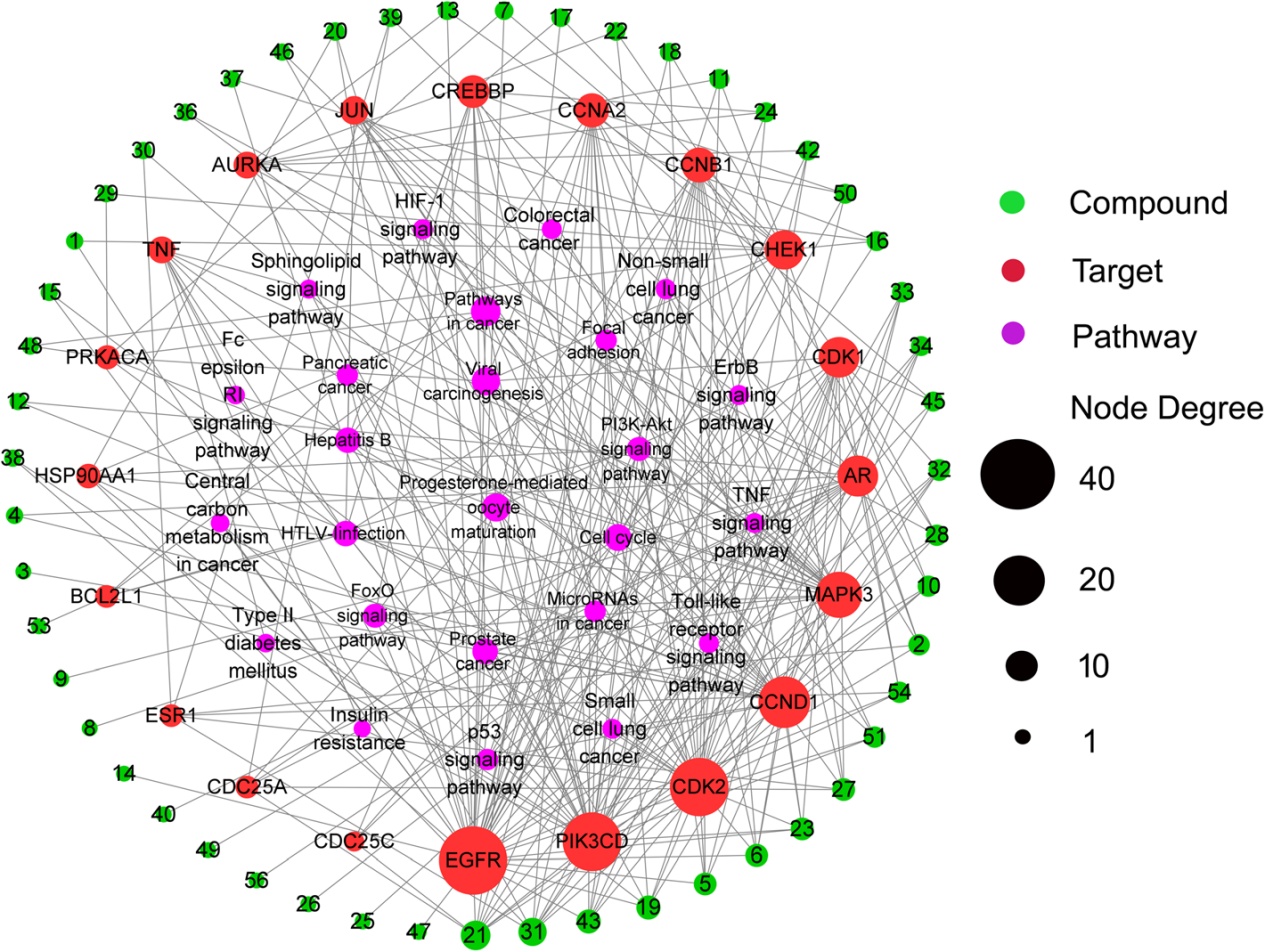
The potential compounds-targets-pathways network of GEB for treating CRC

To explain the mechanism of GEB against CRC, a compound-target pathway network was constructed based on the above compounds, targets, and pathways information. As depicted in Fig. 7, the network was composed of 96 nodes (51 compounds, 20 targets, and 25 pathways) and 315 edges. The green circles, red circles, and pink squares represent active compounds, target proteins, and potential pathways involved in process of GEB against CRC, respectively. The top 3 active compounds with the most degrees are gelegamine E, gelsesyringalidine, and humantenine. The top 3 target proteins with the most degrees are EGFR, PIK3CD, and CDK2.

Red circle nodes stand for the potential target genes related to CRC. Green nodes stand for potential active compounds in GEB for treating CRC. Pink squares stand for pathways. The size of the node represents the number of the degrees.

### Compounds-targets docking

The interaction between components and targets was further evaluated by applying molecular docking, which will validate the accuracy of the compound-target networks analysis.

**Fig. 8:**
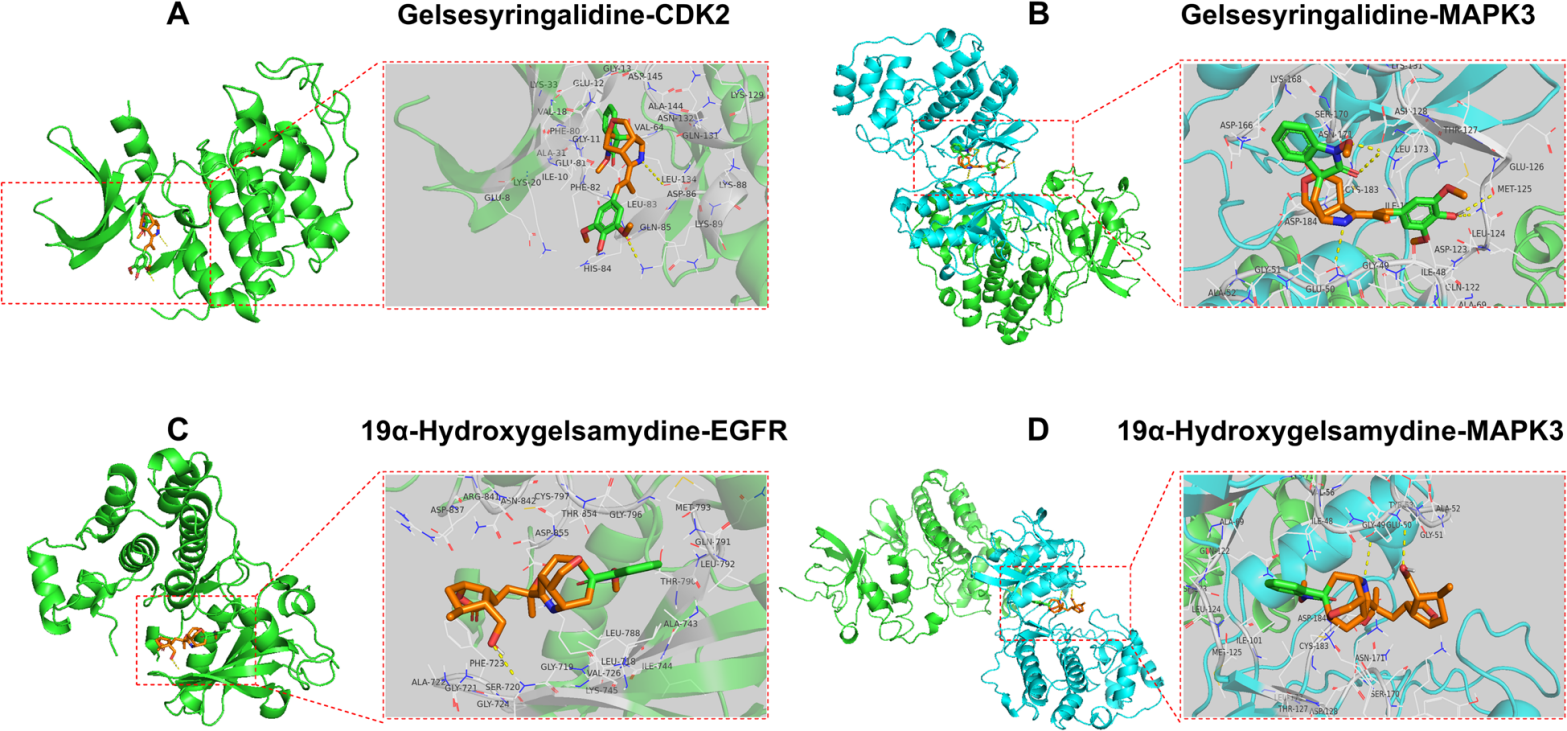
Virtual docking of bioactive compounds of GEB and CRC targets

Virtual screening using AutoDock Vina was performed to calculate the binding affinity between protein models and 10 potentially active compounds (including gelegamine E, gelsesyringalidine, humantenine, 19α-hydroxygelsamydine, gelsedine, 19-Z-akuammidine, gelegamine B, tabersonine, koumine and gelsemine) obtained from compound–target-pathway network. Six targets (EGFR, PIK3CD, CDK2, CCND1, MAPK3, and AR) and 10 compounds were analyzed by molecular docking (Table 3). The top 4 Compound-target dockings with the lowest binding energy were visualized in Fig. 8. The complex of gelsesyringalidine-CDK2 was stabilized by two H-bond with residues including ASP-86 (3.5 Å) and LYS-89 (2.4 Å), the complex of gelsesyringalidine-MAPK3 was stabilized by five H-bond with residues including LYS-131 (2.1 Å, 2.8 Å), TYR-53 (3.2 Å) and MET-125 (2.4 Å, 2.5 Å) (Fig. 8A, B). Meanwhile, 19α-hydroxygelsamydine fixed the binding cavity of CRC target EGFR through one H-bonds with residue including SER-720 (2.6 Å), and hydroxygelsamydine-MAPK3 was stabilized by two H-bonds with residues including TYR-53(3.1 Å) and GLU-50 (2.4 Å) (Fig. 8C, D).

**Table 3:** Virtual docking of ten bioactive compounds of GEB and CRC targets

NO	Name	The number of Hydrogen bond	Amino acid residue	Structure	Target	Binding Energy (kcal·mol^−1^)
21	Gelegamine E	1	LYS`745		EGFR	−8.0
		4	ARG`870		PIK3CD	−5.3
		1	ASP`86		CDK2	−7.7
		2	ARG`59		CCND1	−6.8
31	Gelsesyringalidine	4	ARG`870/GLU`873/SER`878		PIK3CD	−2.9
		2	ASP`86/LYS`89		CDK2	−10.1
		5	LYS`131/MET`125/TYR`53		MAPK3	−9.5
43	Humantenine	NA	NA		EGFR	−8.3
		1	LYS`89		CDK2	−5.7
19	19α-Hydroxygelsamydine	1	ER`720		EGFR	−9.5
		2	TYR`53/GLU`50		MAPK3	−9.7
5	Gelsedine	3	ARG`59/LYS`114		CCND1	−6.7
6	19 - Z- akuammidine	1	ARG`52		CCND1	− 6.6
23	Gelegamine B	4	ARG`870		PIK3CD	−6.3
46	Tabersonine	1	LYS`131		MAPK3	−9.2
36	Koumine	NA	NA		AR	−9.0
		1	SER`170		MAPK3	−7.9
37	Gelsemine	1	CYS`797		EGFR	−8.2

*NA* Not applicable

The virtual docking of gelsesyringalidine with CDK2 and MAPK3 was represented by A and B, respectively. The virtual docking of 19α-Hydroxygelsamydine with EGFR and MAPK3 was represented by C and D, respectively.

### Molecular dynamics simulation

**Fig. 9:**
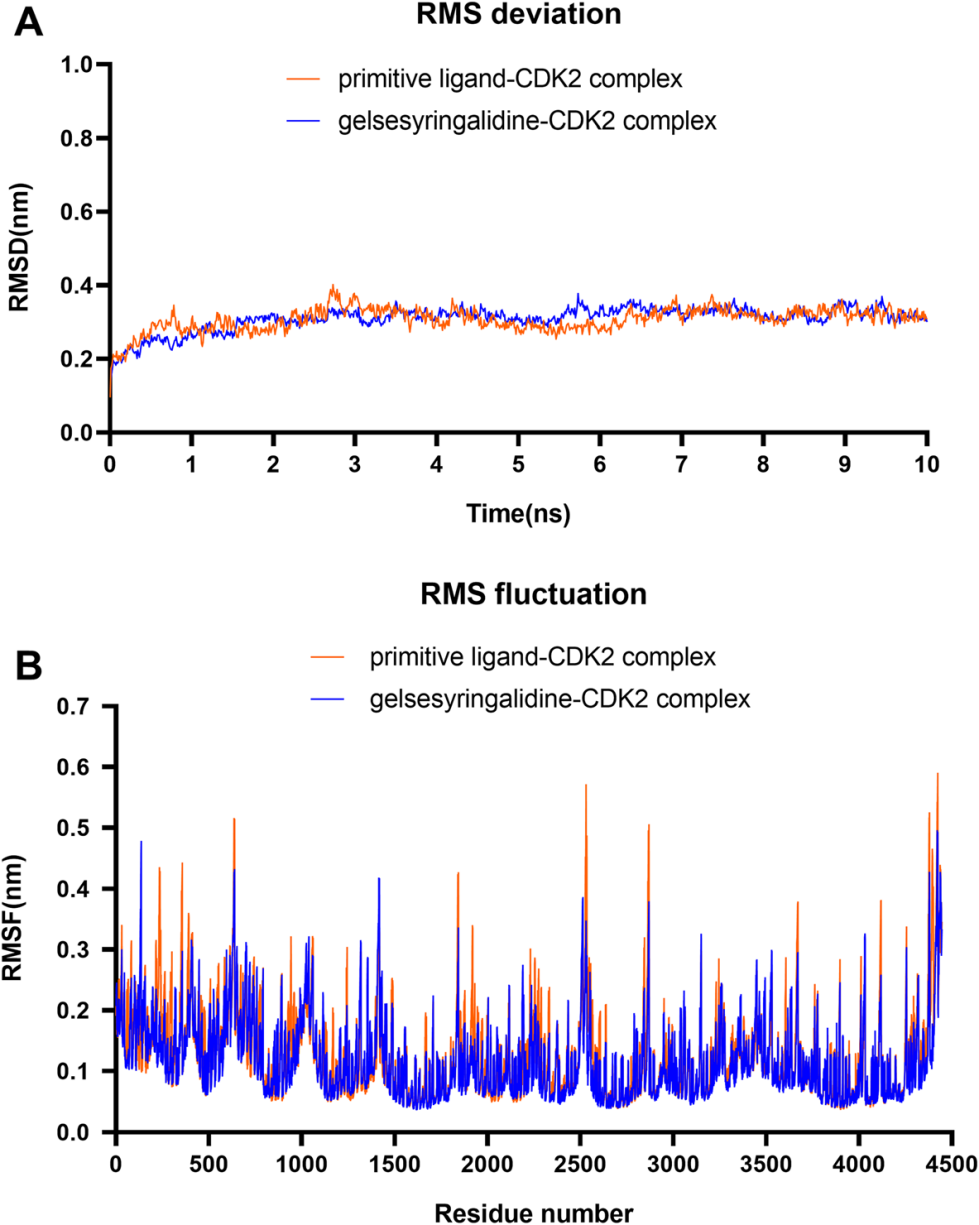
Molecular dynamics simulation of compound-target complex

Molecular dynamics simulation provides a significant insight about the stability of protein-ligand complex. As shown in Fig. 9, the best hit molecules after docking, gelsesyringalidine-CDK2 binding complex showed similar RMSD and RMSF value in comparison to primitive ligand complex AJR-CDK2 binding complex. This observation indicates that gelsesyringalidine forms a stable protein-ligand complexe and does not make any considerable conformational change in the protein structure during simulation.

RMSD and RMSF profiles of ligand-protein complex were represented by A and B, respectively. ARJ, primitive ligand of CDK2: 2,2′-{[6-{[(4-methoxyphenyl)methyl]amino}-9-(propan-2-yl)-9H-purin-2-yl]azanediyl}di(ethan-1-ol).

## Discussion

The Chinese name of GEB is “Duan chang cao”, which literally means a plant can lead to the intestines broken. On the one hand, it shows that this plant has strong toxicity, on the other hand, it may imply a potential target site related to the digestive tract. In China, GEB is used in folk for the treatment of different diseases, including cancer [18]. Consistently, GEB is thought to have anti-cancer properties and exhibit pharmaceutical potential. However, its clinical use is hampered by its toxicity. The prediction of the toxicological hazard when administered orally from the molecular structure of compounds of GEB were evaluated by Toxtree (Table S8). Clinical application of GEB has not been officially approved and is now only used in folk. Despite the high toxicity of GEB and its crude alkaloidal extraction, some monomeric alkaloids of GEB are relatively low in toxicity [16]. For instance, koumine (4.8 mg/kg, intraperitoneally) exhibited significant antitumor activity on mice bearing solid tumor [38]. Whereas, LD50 of koumine is 100 mg/kg (mice, intraperitoneally) [16], demonstrating a high therapeutic index in the treatment of cancer. Hence, GEB may provide a promising number of molecules with proven cytotoxic and apoptogenic activities against CRC, GEB and its alkaloidal components have been abstracting increasing attention for development of antineoplastic drugs [22, 39, 40].

However, the multiple targets, pathways, and mechanisms of its antitumor effect remain unclear. Network pharmacology has been widely adopted by many studies to investigate the potential activity, targets, and pathways of medicinal plants, herb pairs, or herbal formulas with complex ingredients [41, 42]. Thus, network pharmacology provides a novel opportunity for us to investigate potentially pharmacological and molecular mechanisms of GEB against CRC in this study. To the best of our knowledge, this is the first study applying network pharmacology analyses to reveal the pharmacological mechanisms of GEB for treating CRC.

In the present study, a total of 56 compounds of GEB were included in the network pharmacology-based analysis, 53 active compounds were identified with 136 potential targets related to CRC. Many targets were discovered to be hit by multiple compounds. For example, the top 3 targets, EGFR, IGF1R, and ABCB1 were modulated by more than 20 ingredients. Also, ingredients of GEB such as gelsesyringalidine, Gelegamine E, and hydroxygenkwanin can regulate more than 30 targets. This fact indicated that the bioactive ingredients of GEB might regulate multiple targets and can affect these targets synergistically. Therefore, active ingredients of GEB have therapeutic effects not only on CRC but also on other diseases, which virtually confirmed the nature of multicomponent, multi-target, and multi-disease of plant medicine. Hence, we could not only acquire valuable information on the relationship between active ingredients and its potential targets but also discover the other potential effects of GEB from the network pharmacology-based analysis.

PPI analysis of 136 targets revealed that the top 20 of center target genes, were MAPK3, HSP90AA1, JUN, EGFR, CDK1, TNF, CCND1, ESR1, PRKACA, CCNA2, CDC25C, CDK2, CCNB1, AR, CREBBP, AURKA, CDC25A, CHEK1, BCL2L1, and PIK3CD, which were likely to be crucial targets for GEB in treating CRC. GEB may exert its therapeutic effect against CRC by regulating these specific protein targets. Consider MAPK3, HSP90AA1, JUN, EGFR, CDK1. MAPK3 was simultaneously targeted by 4 active chemicals: 19α-hydroxygelsamydine, gelsesyringalidine, koumine, and tabersonine. Mitogen-activated protein kinase 3 (MAPK3) belongs to the protein kinase superfamily and catalyzes the concomitant phosphorylation of a threonine and a tyrosine residue in the MAP kinase p38. It has been confirmed to be a potential therapeutic target for different kinds of human cancers, including CRC [43–47]. A previous study revealed that phosphorylated AMP-activated protein kinase (AMPK) expression in CRC was associated with superior prognosis among p-MAPK3 positive cases, indicating a possible interaction between the AMPK and MAPK pathways influencing tumor behavior [46]. HSP90AA1, a heat shock protein HSP 90-alpha, promotes the maturation, structural maintenance, and proper regulation of particular target proteins involved in signal transduction and cell cycle control. HSP90AA1 is tightly related to gastrointestinal cancers, such as esophageal, gastric, and colon cancers, and can be predictive biomarkers for these cancers [48]. As for JUN, it is known as transcription factor AP-1, is involved in activated KRAS-mediated transcriptional activation of USP28 by binding to the USP28 promoter in CRC cells. Its mutation in the promoter region is associated with increased CRC risk by elevating promoter activity [49]. JUN plays a key role in regulating and promoting the signaling pathways related to carcinogenesis, cell proliferation, metabolism, migration, apoptosis, and survival [50–52]. EGFR, the ErbB family of related cell membrane receptors, is a receptor tyrosine kinase binding ligand of the EGF family. EGFR family is associated with anti-apoptosis, proliferation, metastasis, and drug resistance in CRC, making this pathway a particularly compelling target for drug design [53]. Notably, EGFR was predicted to correlate with the most active ingredients (28 of 53) of GEB, suggesting that these active components of GEB may exert therapeutic effects synergistically via regulating EGFR expression or its function. CDK1 plays a crucial role in controlling the eukaryotic cell cycle by modulating the centrosome cycle as well as the mitotic onset. It promotes G2-M transition and regulates G1 progress and G1-S transition via association with multiple interphase cyclins. Accordingly, expression of CDK1 has been demonstrated to be enhanced in CRC [54, 55]. Though the study on the molecular mechanism of anti-cancer action of GEB is limited, fortunately, consistent with the prediction of our study, a previous research has shown that koumine, the most abundant ingredient in alkaloidal components of GEB, suppressed hepatocellular carcinoma cell proliferation via MAPK signaling pathway [56]. Furthermore, the compounds-targets docking analysis results also demonstrated that there was good affinity between MAPK3 and several compounds of GEB including koumine, confirming the role of MAPK3 as one of the key targets in the anti-cancer effect of GEB. On the whole, our result suggested that active compounds of GEB might produce anti-cancer effects by interacting with these key targets.

In order to better understand the multiple mechanisms of GEB against CRC from a systematic point of view, we performed a GO enrichment analysis of the 136 selected targets, consisting of the biological processes, molecular functions, and cellular components. Functional enrichment analysis revealed the over-represented GO terms and their functional domains. The top 10 GO functional categories were shown in Fig. 6, these demonstrated that GEB may produce its effect by involving in the above biological processes, molecular functions, and cellular components. Based on GO enrichment analysis, BP terms enriched by target genes were mainly concentrated in response to various phosphorylation (GO:0018108: peptidyl-tyrosine phosphorylation, GO:0046777: protein autophosphorylation, GO:0006468: protein phosphorylation, GO:0001934: positive regulation of protein phosphorylation, and GO:0018105: peptidyl-serine phosphorylation). Protein phosphorylation is a pivotal cellular regulatory mechanism as many enzymes and receptors are activated/deactivated by phosphorylation, which play a key role in the control of biological processes such as proliferation, differentiation and apoptosis [57]. Positive regulation of cell proliferation (GO:0008284) and MAPK cascade (GO:0043410) are also important in colorectal tumorigenesis [58]. Correspondingly, MF terms were strongly correlated with different kinases activity, such as transmembrane receptor protein tyrosine kinase activity (GO:0004714), protein kinase activity (GO:0004672), and protein tyrosine kinase activity (GO:0004713). The functional enrichment analysis implied that GEB might exhibit its anti-CRC effect by regulating of transcription, such as the different kinases activity, resulting in phosphorylation change in cell signaling pathway.

KEGG pathway enrichment analysis revealed that the 136 target proteins were significantly enriched in 70 related signaling pathways. In light of the results of these well-known cancer-related pathway enrichment, we believe GEB can simultaneously target multiple pathways pathways. Among 70 signaling pathways we obtained, colorectal cancer(hsa05210) is the most crucial one that exerts regulatory effect on the process of genetic stability, proliferation, apoptosis, and survival of CRC cells. Furthermore, we found that GEB may exert a therapeutic effect against CRC through other multiple signaling pathways. For instance, FoxO pathway(hsa04068) are involved in cell cycle regulation and proliferation process, activation of FoxO signaling pathway can induce apoptosis effect on human CRC [59]. The activation of the PI3K/AKT pathway(hsa04151) is known to have an important role in the development and progression of CRC, PI3K/AKT signaling leads to reduced apoptosis, stimulates cell growth and increases proliferation [60]. Tumor protein p53 as a well-known transcription factor and tumor suppresser, regulates the expression of a wide variety of genes involved in apoptosis, growth arrest, or senescence in response to genotoxic or cellular stress. Hence, inactivation of the p53 pathway is often observed in CRC [61, 62]. MicroRNAs in cancer (hsa05206) participate in tumorigenesis, progression, invasion, and drug resistance in different cancers, including CRC [63]. Chronic inflammation is one of the characteristics of CRC. Tumor necrosis factor alpha (TNF-α) mediates the inflammatory response, which can activate signal transducer and activator of transcription 3 (STAT3), nuclear factor and kappa-B (NF-κB), resulting in progression of CRC [64]. Yuan reported that koumine could promote ROS production to suppress hepatocellular carcinoma cell proliferation Via NF-kappaB signaling and attenuate lipopolysaccaride-stimulated inflammation in RAW264.7 macrophages, coincidentally associated with inhibition of NF-kappaB pathways [56, 65]. These findings may patially support our prediction on KEGG pathway enrichment analysis. To validate prediction of these pathways, microarray technology like Affymetrix GeneChip, may be employed to analysis target gene expression after the treatment of GEB or its key ingredients in future pathway study. Meanwhile, we also found that some signaling pathways significantly enriched by targets were closely related to other cancers, indicating that GEB might exert effects on various malignant tumors, like prostate cancer, lung cancer, and pancreatic cancer.

Total of 53 active compounds were identified by the network pharmacology-based approach. According to Compound-target-pathway network analysis, gelegamine E, gelsesyringalidine, and humantenine were among top 3 key ingredients with the highest degrees in targets. However, they are not the most abundant ingredients in GEB. Hereto, there is no report regarding their pharmacological activities because of their accessibility. With the help of our previous established method of pH-zone-refining counter-current chromatography [66], more monomers from GEB are hopefully available and their anti-CRC effect will be identified. As the most abundant monomer of GEB, Koumine has been attracting much attention in recent decade. Encouragingly, consistent with our prediction, koumine was proved to induce apoptosis of the human colon adenocarcinoma LoVo and SW480 cells [40, 67],as well as human hepatoma Bel7402 and H22 cells [38]. In vivo study, koumine also exhibited anticancer effect that comparable with 5-fluorouracil in the model of mice bearing the hepatoma cancer [67]. Moreover, koumine is relatively low in toxicity compared with other components, like gelsemine [16, 68]. Hence, it is promising to discover more novel antineoplastic monomers in GBE with low toxicity based on network pharmacology analysis.

## Conclusion

In this study, based on network pharmacology analysis, we obtained 53 active compounds from GEB and predicted 20 potential center targets for GEB in the treatment of CRC, suggesting that GEB was an herbal medicine with multicomponent, multiple targets, and multiple pathways. The network analysis revealed that GEB may exert its therapeutic effects against CRC by modulating certain distinct targets, such as MAPK3, HSP90AA1, JUN, EGFR, CDK1, TNF CCND1, ESR1, PRKACA, and CCNA2. The GO analysis of these targets demonstrated that the compounds of GEB likely produced pharmacological effects against CRC mainly by influencing different biological processes, like regulation of peptidyl-tyrosine phosphorylation, protein autophosphorylation, and protein phosphorylation. Meanwhile, the KEGG pathway analysis in the present study disclosed that GEB probably exerted its pharmacological action via simultaneously regulating different signaling pathways related to CRC, such as colorectal cancer, pathways in cancer, FoxO signaling pathway, and PI3K-AKT signaling pathway.

To summarize, the present study is the first one that undertakes a network pharmacology- based analysis to explore the potential pharmacological and molecular mechanism of GEB in CRC treatment from a systematic point of view. The results indicated that GEB could be a promising agent in the treatment for CRC with multiple components, targets, and pathways. Our study also provides a theoretical basis for the further development of GEB in the future. However, as this study was based on data mining and data analysis, and there is limited study on the GEB against CRC, more validated experiments are warranted to verify our prediction.

## Supplementary Information

**Additional file 1.**

## Data Availability

All data generated or analysed during this study are included in this published article and its supplementary information files.

## Abbreviations

CRC: Colorectal cancer
GEB: *Gelsemium elegans Benth*
TCM: Traditional Chinese Medicine
TCMID: Traditional Chinese Medicines Integrated Database
BATMAN-TCM: Tool for Molecular mechanism of Traditional Chinese Medicine
DAVID: Database for annotation, visualization and integrated discovery
GO: Gene Ontology
KEGG: Kyoto Encyclopedia of Genes and Genome
TCGA: Cancer Genome Atlas
PPI: Protein-Protein Interaction
MCODE: Molecular Complex Detection
FDR: False Discovery Rate
BP: Biological process
CC: Cellular component
MF: Molecular function
NPTS: Number of grid points in the three dimensions
MAPK3: Mitogen-activated protein kinase 3
AMPK: Phosphorylated AMP-activated protein kinase
